# A Diagnostic Odyssey: Mevalonate Kinase Deficiency Revealed by Genetic Testing in Adulthood

**DOI:** 10.3390/genes17040439

**Published:** 2026-04-11

**Authors:** Vijayalakshmi Kumar, Constance Jensina de Saint-Aubain, Konstantin N. Konstantinov

**Affiliations:** 1 Medicine Service, Section of Rheumatology, Raymond G. Murphy VA Medical Center, Albuquerque, NM 87108, USA; 2 Division of Rheumatology, Department of Medicine, University of New Mexico, Albuquerque, NM 87131, USA; 3 Sundets Data Science, P.O. Box 2970 Copenhagen, Denmark

**Keywords:** mevalonate kinase deficiency, autoinflammatory diseases, hyperimmunoglobulinemia D, periodic fever syndromes

## Abstract

Mevalonate kinase deficiency (MKD) is a rare autosomal recessive autoinflammatory disorder with marked clinical heterogeneity, frequently leading to delayed diagnosis. We describe a 71-year-old woman with lifelong episodic inflammatory symptoms beginning in infancy, including recurrent fevers, lymphadenopathy, and gastrointestinal and mucocutaneous manifestations, later evolving into intermittent arthralgia, myalgia, and fatigue. A unifying diagnosis was established when genetic testing identified two missense pathogenic *MVK* variants consistent with compound heterozygous MKD, supported by elevated serum IgD levels and a characteristic clinical phenotype. This case illustrates the essential role of molecular genetic testing in resolving prolonged diagnostic odyssey, in guiding surveillance for complications such as AA amyloidosis, and enabling targeted therapeutic strategies.

## 1. Introduction

Autoinflammation and autoimmunity were originally viewed as mechanistically distinct immune disorders, driven primarily by dysregulation of the innate and adaptive immune systems, respectively. The identification of monogenic autoinflammatory diseases such as familial Mediterranean fever and TNF receptor-associated periodic syndrome established a model of inflammation arising from aberrant innate immune sensing, inflammasome activation, and excessive cytokine release, in the absence of pathogenic autoantibodies or autoreactive lymphocytes [1,2]. In contrast, autoimmune diseases were traditionally defined by a breakdown of adaptive immune tolerance and characterized by the emergence of autoreactive T and B cells and pathogenic autoantibodies. However, this binary distinction has become increasingly blurred. Many prototypical autoimmune diseases display strong innate immune signatures—e.g., systemic lupus erythematosus, long regarded as antibody-driven, is now recognized to be critically shaped by complement activation and type I interferon pathways, implicating innate immune amplification as a central driver of disease [3]. Conversely, inflammasome-derived cytokines such as IL-1β and IL-18, which are hallmarks of autoinflammatory disorders, play key roles in shaping adaptive immunity by promoting T-cell survival, B-cell activation, and antibody production [4]. Genetic, experimental, and clinical data therefore support a continuum model in which autoinflammation and autoimmunity represent overlapping, interconnected processes rather than discrete entities, linked by bidirectional crosstalk between innate and adaptive immune pathways [5].

In this report we describe a patient with rare autosomal recessive autoinflammatory disease caused by pathogenic variants in the mevalonate kinase (*MVK)* gene. Defective MVK activity disrupts the isoprenoid biosynthesis pathway, leading to impaired prenylation of small GTPases and exaggerated innate immune activation, most notably through increased interleukin-1β production [6]. IL-1β is not the only cytokine involved in the inflammatory response in MKD. Although classified as an autoinflammatory disease, sustained inflammasome activation with excess IL-1β and IL-18 promotes adaptive immune activation through IL-6-dependent B-cell stimulation, Th1 skewing, and impaired immune tolerance, providing a biologic bridge to secondary autoimmune phenomena.

Mevalonate kinase deficiency (MKD) comprises a spectrum of disorders, ranging from mevalonic aciduria (MA) at the severe end to hyper-IgD and periodic fever syndrome (HIDS) at the milder end. Because clinical manifestations overlap with other hereditary autoinflammatory syndromes, molecular genetic testing has emerged as the diagnostic cornerstone for MKD. Identification of biallelic pathogenic *MVK* variants enables definitive diagnosis, informs prognosis, guides targeted anti-IL-1-based therapy, and facilitates genetic counseling, underscoring the critical role of comprehensive genetic testing in the evaluation of patients with suspected MKD/HIDS.

## 2. Clinical Vignette

A 71-year-old woman was referred to rheumatology for evaluation of lifelong episodic inflammatory symptoms. On careful history, the patient recalls recurrent high fevers beginning in infancy, often triggered by minor infections or childhood vaccinations, accompanied by painful cervical lymphadenopathy, abdominal pain with diarrhea, oral aphthous ulcers, and intermittent erythematous rashes. During childhood, these episodes occurred with predictable regularity and were associated with markedly elevated inflammatory markers, though extensive infectious and autoimmune evaluations were unrevealing. Over time, the frequency and intensity of attacks gradually diminished, and by adolescence the febrile episodes became less frequent, presenting instead with episodic arthralgia, myalgia, and profound fatigue. Review of prior records reveals persistently elevated acute-phase reactants during flares and intermittent elevation of serum IgD. In adulthood, symptoms were sporadic but relapsing, prompting renewed evaluation.

A genetic test (Invitae Periodic Fever Syndrome Panel (Labcorp Genetics/Invitae, San Juan Capistrano, CA, USA) using next-generation sequencing covering coding regions, flanking introns, and some non-coding areas revealed two missense pathogenic heterozygous variants in the *MVK* gene, the *MVK* variant c.1129G>A (p.Val377Ile) and the *MVK* variant 803T>C (p.Ile268Thr). Serum IgD was elevated at 363 mg/L (reference range: <179 mg/L). These results together with the constellation of clinical symptoms are consistent with those of MKD/HIDS in our patient.

Following genetic confirmation of MKD, therapy was tailored to the patient’s current phenotype, marked by rare, transient flares with low inflammatory burden and no evidence of end-organ involvement. At present the patient is managed with on-demand anti-inflammatory therapy during mild flares (NSAIDs as needed) without immediate initiation of biologic therapy. The decision to defer IL-1 blockage was guided by the patient’s wishes, advance age, and risk–benefit considerations as well. A structured monitoring strategy was implemented, which included periodic assessment of inflammatory markers (CRP, ESR, and serum amyloid A), renal function, and surveillance for proteinuria, given the risk of AA amyloidosis. Establishing a molecular diagnosis after decades of unexplained inflammation had immediate clinical implications: it terminated diagnostic uncertainty, enabled pathway-specific therapeutic planning, and prompted prospective risk stratification. Importantly, it reframed management from empiric immunosuppression to precision surveillance with a low threshold for IL-1-targeted therapy.

## 3. Chronology of MKV Discovery

Mevalonate kinase (MK) catalyzes the phosphorylation of mevalonate to mevalonate 5-phosphate, a key step in isoprenoid and cholesterol biosynthesis [7]. In humans, it is ubiquitously expressed, notably in leukocytes and keratinocytes. MVK, the gene encoding MK, is located on chromosome 12q24 and contains ten coding exons and one non-coding exon [8].

While the enzyme was studied earlier, the genetic basis of MVK started with gene cloning in 1992 and the identification of disease-causing mutations in 1997 [9]. Several years later, MVK variants were linked to a distinct periodic fever syndrome with high serum IgD, described in the mid-1980s and later called hyper-IgD/periodic fever syndrome (HIDS) [10]. Subsequent studies of larger groups of HIDS patients with dysregulated, increased IgD levels showed that it is not a constant or reliable feature and may be an effect of the inflammatory reaction, rather than its cause [11].

## 4. Mode of Inheritance and Disease Frequency

MKD follows an autosomal recessive inheritance pattern, which implies that the pathogenic defect affects both MVK alleles, one from each parent. Two pathogenic heterozygous mutations in *trans*, known as compound heterozygous MKD genotype, also support the diagnosis of MKD, provided the clinical picture is compatible. In contrast, a carrier has only one mutation and is usually asymptomatic. Nevertheless, cases with MKD have been reported [12] in which absence of a second detectable pathogenic *MVK* variant does not exclude recessive disease, when transcriptional silencing or structural alterations lead to undetected loss of allele expression. The resulting loss of *MVK* expression from one allele, combined with a pathogenic variant on the expressed allele, produces a biallelic functional deficiency that fulfills the biologic requirement for disease despite incomplete genotypic detection. Currently, 285 sequence variants for *MVK* have been listed on the Registry of Hereditary Autoinflammatory Disorders Mutations (Infevers website, editor, Lawrence Cuisset; https://infevers.umai-montpellier.fr/web/search.php?n=3, accessed on 20 February 2026).

Fewer than 300 patients with MKD have been reported worldwide in the literature [13,14]. However, this is likely an underestimate of the true population, as many cases remain undiagnosed or underreported due to the condition’s rarity and variable presentation.

## 5. Most Common Variants

Across published cohorts, MKD is dominated by two recessively inherited missense mutations in compound heterozygosity or homozygous state. Recurrently reported variants include p.V377I (1129G>A)—the most common variant causing MVK defect in European patients [15] that can also be associated with p.I268T (c.803T>C) [12]. The p.V377I variant primarily affects protein stability and folding, rather than catalytic function, preserving some residual normal mevalonate kinase activity [16]. The second most common mutation in MKD/HIDS patients is the p.I268T variant. This variant causes more pronounced enzymatic impairment, disrupting both protein stability and catalytic function [17]. In many patients with MKD, a hypomorphic missense variant in one allele allows for residual MVK enzyme activity, which is associated with less severe clinical disease (e.g., HIDS) compared with variants that cause near-complete loss of function and mevalonic aciduria [16].

The p.Val377Ile/p.Ile268Thr genotype is one of the most common combinations in MKD/HIDS. In the Eurofever Registry, AA amyloidosis was significantly associated with the p.V377I/p.I268T compound heterozygous genotype. Also, patients who lacked a p.V377I mutation had more severe musculoskeletal involvement [18]. However, genotype–phenotype correlations demonstrate considerable variability, with clinical heterogeneity observed even among patients with identical genotypes, suggestion influence of modifying factors, including genetic modifiers, environmental conditions, and individual metabolic variation [19].

## 6. Clinical Phenotypes of Mevalonate Kinase—Associated Disease

The symptomatic spectrum of MKD presents with variable clinical phenotypes. The most extreme form is known as mevalonic aciduria, which in addition to recurrent fevers presents with developmental delays, facial dysmorphia, cerebellar ataxia, enlarged organs, and in some cases premature death [20]. The mild presentation, known as hyperimmunoglobulinemia D and periodic fever syndrome (HIDS), is characterized predominantly by recurrent fever with inflammatory symptoms. Importantly, these entities do not represent discrete categories; rather, MKD exists along a continuum, reflecting downstream effects of *MVK* mutations and variable residual mevalonate kinase enzymatic activity that give rise to graded clinical severity [21].

The clinical spectrum of MKD phenotypes has continued to expand. Recently, *MVK* mutations have been associated with disseminated superficial actinic porokeratosis (DSAP), a disease characterized by multiple, small keratotic lesions on sun-exposed skin. PK comprises a group of predominantly autosomal conditions, most reported in individuals of Asian ancestry [22]. In DSAP, no abnormal IgD serum levels have been described. To date, porokeratotic lesions have not been described in patients with MKD, and the observation that identical mutations can underlie both systemic inflammatory and isolated cutaneous phenotypes remains puzzling. Other examples of MKD clinical heterogeneity are increasingly reported case presentations describing immune-mediated end-organ damage. Emerging atypical associations include links between mevalonic aciduria and retinitis pigmentosa [23], as well as MKD presenting with early-onset inflammatory bowel disease [24], prominent liver involvement [25] and cryptogenic ischemic stroke [26].

## 7. Value of Correct Diagnosis

Getting the right diagnosis is a key aspect of health care. When diagnosis is delayed, patients often require prolonged treatments, additional tests, hospitalizations, and increased costs. Certain conditions are particularly susceptible to diagnostic delays because of their rarity, complex nature, or subtle and nonspecific presentation. In our case, diagnostic delay was probably due to a combination of factors: nonspecific nature of symptoms, limited access to specialized care, and, until recently, the lack of widely available genetic testing. Advances in genetic testing reduce diagnostic uncertainty by providing precise identification of inherited disorders and offer personalized approaches to risks stratification and treatment. In our patient, genetic testing identified pathogenic variants in the *MVK* gene, establishing the diagnosis of MKD and reframing decades of episodic inflammation. It should also be noted that diagnostic delay is not unusual. In a French and Belgian cohort, the diagnosis of MKD was reached on average 37 years after symptom onset [19].

Diagnosis is now primarily established by identifying biallelic pathogenic variants in the *MVK* gene, with genetic testing serving as the gold standard. Urinary mevalonic acid may support the diagnosis—particularly during febrile attacks—but has limited sensitivity and may be normal between episodes; thus, a normal result does not exclude MKD.

Modern management of MKD is IL-1-centric, with canakinumab or anakinra as first-line therapy, supported by selective use of TNF or IL-6 blockade in refractory disease. Early recognition and targeted treatment substantially reduce disease burden and diagnostic delay [21]. Although this patient has not yet required IL-1β therapy, given the decline in flare activity in recent years, she remains under close surveillance for secondary AA amyloidosis, which is a known, rare, complication of MKD/HIDS.

## 8. Conclusions

Our case illustrates the extraordinary diagnostic latency of mevalonate kinase deficiency (MKD), spanning seven decades, and representing one of the longest reported intervals between symptom onset and definitive diagnosis. Although delayed recognition is characteristic of MKD [19], diagnosis in the seventh decade of life remains exceptionally rare and reflects the marked phenotypic heterogeneity and attenuation with age, misclassification as infection/autoimmune disease and lack of access to genetic testing.

A central feature of the prolonged clinical presentation in our patient is the transition from a classical early-life autoinflammatory phenotype to an attenuated adult presentation, dominated by arthralgia, myalgia, and fatigue. This supports the concept that MKD exists along a lifelong continuum in which diminished inflammatory intensity may obscure underlying monogenic process. The case further highlights a key clinical lesson: monogenic autoinflammatory diseases should be considered across the lifespan, and genetic testing can reveal actionable diagnoses even later in life.

## Data Availability

No new data were generated or analyzed in this study. The narrative review is based on publicly available information, and references cited are available through the referenced journals or publishers.

## References

[1] The French FMF Consortium (1997). A candidate gene for the familial Mediterranean fever. Nat. Genet..

[2] McDermott M.F., Aksentievich I., Galon J., McDermott E.M., Ogunkolade B.W., Centola M., Mansfield E., Gadina M., Karenko L., Pettersson T. (1999). Gerline mutations in the extracellular domains of the 55 kDa TNF receptor, TNFR1, define a family of dominantly inherited autoinflammatory syndromes. Cell.

[3] Liu A., Su Y., Zhu J., Li Y.-Y. (2025). Disease association study of autoimmune and autoinflammatory diseases by integrating multi-modal data and hierarchical ontologies. Front. Immunol..

[4] Ke Q., Greenwalt A.N., Manukonda V., Ji X., Tisch R.M. (2023). The regulation of self-tolerance and the role of inflammasome molecules. Front. Immunol..

[5] Meertens B., Hoste L., Tavernier S.J., Haerymck F. (2024). The riddle of recurrent fever: A clinical approach to pediatric autoinflammatory diseases. Front Pediatr..

[6] Mulders-Manders C.M., Simon A. (2015). Hyper-IgD syndrome/mevalonate kinase deficiency: What is new?. Semin. Immunopathol..

[7] Schafer B.L., Bishop R.W., Kratunis V.J., Kalinowski S.S., Mosley S.T., Gibson K.M., Tanaka R.D. (1992). Molecular cloning of human mevalonate kinase and identification of a missense mutation in the genetic diseases mevalonic aciduria. J. Biol. Chem..

[8] Drenth J.P., Cuisset L., Grateau G., Vasseur C., van de Velde-Visser S.D., de Jong J.G., Beckmann J.S., van der Meer J.W., Delpech M. (1999). Mutations in the gene encoding mevalonate kinase cause hyper-IgD and periodic fever syndrome. International hyper-IgD study group. Nat. Genet..

[9] Touitou I. (2022). Twists and turns of the genetic story of mevalonate kinase-associated diseases: A review. Genes Dis..

[10] Van Der Meer J.M., Radl J., Meyer C.L., Vossen J., Van Nieuwkoop J., Lobatto S., Van Furth R. (1984). Hyperimmunoglobulinaemia D and periodic fever: A new syndrome. Lancet.

[11] Ammouri W., Cuisset L., Rouaghe S., Rolland M.-O., Delpech M., Grateau G., Ravet N. (2007). Diagnostic value of serum immunoglobulinaemia D level in patients with clinical suspicion of hyper IgD syndrome. Rheumatology.

[12] Cuisset L., Drenth J.P., Simon A., Vincent M.F., van der Velde Visser S., van der Meer J., Grateau G., Delpech M. (2001). International Hyper-IgD Study Group. Molecular analysis of MVK mutations and enzymatic activity in hyper-IgD and periodic fever syndrome. Eur. J. Hum. Genet..

[13] Zhang S. (2016). Natural history of mevalonate kinase deficiency: A literature review. Pediatr. Rheumatol..

[14] Govindaraj G.M., Jain A., Peethambaran G., Bhoyar R.C., Vellarikkal S.K., Ganapati A., Sandhya P., Edavazhippurath A., Dhanasooraj D., Puthenpurayil J.M. (2020). Spectrum of clinical features and genetic variants in mevalonate kinase (MVK) gene of South Indian families suffering from hyperimmunoglobulin D syndrome. PLoS ONE.

[15] Houten S.M., van Woerden C.S., Wijburg F.A., Wanders R.J.A., Waterham H.R. (2003). Carrier frequency of the V377I (1129G>A) MVK mutation, associated with hyper-IgD and periodic fever syndrome, in the Netherlands. Eur. J. Hum. Genet..

[16] Mandey S.H.L., Schneiders M.S., Koster J., Waterham H.R. (2006). Mutational spectrum and genotype-phenotype correlations in mevalonate kinase deficiency. Hum. Mutat..

[17] Fu Z., Wang M., Miziorko H.M., Kim J.J.P. (2002). The Structure of a Binary Complex between a Mammalian Mevalonate Kinase and ATP: Insights into the Reaction Mechanism and Human Inherited Disease. J. Biol. Chem..

[18] Ter Haar N.M., Jeyaratnam J., Lachmann H.J., Simon A., Brogan P.A., Doglio M., Cattalini M., Anton J., Modesto C., Quartier P. (2016). The Phenotype and Genotype of Mevalonate Kinase Deficiency: A Series of 114 Cases From the Eurofever Registry. Arthritis Rheumatol..

[19] Durel C.A., Aouba A., Bienvenu B., Deshayes S., Coppéré B., Gombert B., Acquaviva-Bourdain C., Hachulla E., Lecomte F., Touitou I. (2016). Observational Study of a French and Belgian Multicenter Cohort of 23 Patients Diagnosed in Adulthood With Mevalonate Kinase Deficiency. Medicine.

[20] Jeyaratnam J., ter Haar N.M., de Sain-van der Velden M.G.M., Waterham H.R., van Gijn M.E., Frenkel J. (2016). Diagnostic value of urinary mevalonic acid excretion in patients with a clinical suspicion of mevalonate kinase deficiency. JIMD Rep..

[21] Lengvari L., Lengyel A., Pálinkás A., Wouters C.H., Kone-Paut I., Kuemmerle-Deschner J., Jeyaratnam J., Anton J., Lachmann H.J., Gattorno M. (2024). Mevalonate kinase deficiency: An updated clinical overview and revision of the share recommendations. Front. Immunol..

[22] Zhang Z., Li C., Wu F., Ma R., Luan J., Yang F., Liu W., Wang L., Zhang S., Liu Y. (2015). Genomic variations of the mevalonate pathway in porokeratosis. Elife.

[23] Siemiatkowska A.M., Van Den Born L.I., Van Hagen P.M., Stoffels M., Neveling K., Henkes A., Kipping-Geertsema M., Hoefsloot L.H., Hoyng C.B., Simon A. (2013). Mutations in the mevalonate kinase (MVK) gene cause nonsyndromic retinitis pigmentosa. Ophthalmology.

[24] Bianco A.M., Girardelli M., Vozzi D., Crovella S., Kleiner G., Marcuzzi A. (2014). Mevalonate kinase deficiency and IBD: Shared genetic background. Gut.

[25] Hinson D.D., Rogers Z.R., Hoffmann G.F., Schachtele M., Fingerhut R., Kohlschutter A., Kelley R.I., Gibson K.M. (1998). Hematological abnormalities and cholestatic liver disease in two patients with mevalonate kinase deficiency. Am. J. Med. Genet..

[26] Hamidi L.-N., Drda J.C., Belhocine M., Elfassy H.-L., Ducharme-Benard S., Chayer-Lanthier M., Suktana B., Lanthier S. (2025). Case report: Mevalonate kinase deficiency: An underdiagnosed cause of ischemic stroke—characterization of a novel genetic variant. Front. Immunol..

